# Low back pain during pregnancy caused by a sacral stress fracture: a case report

**DOI:** 10.1186/1752-1947-6-98

**Published:** 2012-04-04

**Authors:** Miguel Pishnamaz, Richard Sellei, Roman Pfeifer, Philipp Lichte, Hans C Pape, Philipp Kobbe

**Affiliations:** 1 Department of Orthopedic and Trauma Surgery, University of Aachen Medical Center, 30 Pauwels Street, 52074 Aachen, Germany

## Abstract

**Introduction:**

Sacral stress fractures are a rare but well known cause of low back pain. This type of fracture has also been observed as a postpartum complication. To date, no cases of intrapartum sacral stress fractures have been described in the literature.

**Case presentation:**

We report the case of a 26-year-old Caucasian European primigravid patient (30 weeks and two days of gestation) who presented to our outpatient clinic with severe low back pain that had started after a downhill walk 14 days previously. She had no history of trauma. A magnetic resonance imaging scan revealed a non-displaced stress fracture of the right lateral mass of her sacrum. Following her decision to opt for non-operative treatment, our patient received an epidural catheter for pain control. The remaining course of her pregnancy was uneventful and our patient gave birth to a healthy child by normal vaginal delivery.

**Conclusions:**

We conclude that a sacral stress fracture must be considered as a possible cause of low back pain during pregnancy.

## Introduction

Although sacral stress fractures are uncommon, they are a well-known cause of low back pain, especially in athletes [1-5]. The underlying pathology of stress fractures is either a weakened bone or an unusually high load that normal bone is unable to withstand [6]. The latter has been cited by several authors as the cause of postpartum sacral fractures occurring during the course of childbirth [7-10]. Usually, the fracture is diagnosed by magnetic resonance imaging (MRI), which shows a vertical fracture line with surrounding osseous edema. This fracture is particularly challenging during pregnancy since adequate analgesic control is complicated by drug interactions with the fetus, as well as the continued load imposing stress on the sacrum over the course of the pregnancy. To the best of our knowledge, this case is the first report of an intrapartum sacral stress fracture and outlines the difficulties and limitations of pain control in these patients.

## Case presentation

A 26-year-old Caucasian European primigravid patient (30 weeks plus two days of gestation) was transferred from a local area hospital with severe low back pain. She first experienced slight discomfort over her bilateral iliosacral joints while walking downhill 14 days previously. She reported that water exercises gave her complete pain relief. Three days after the initial discomfort, she exacerbated the condition and experienced severe pain while exiting the passenger seat of her car. The pain was described as radiating from the right side of her lower back to the back side of her knee, and being electric in character. At that point, she was unable to walk independently and was admitted to the hospital for pain management with oral paracetamol.

On admission to our hospital, a clinical examination revealed a healthy young woman with a height of 162 cm and a weight of 56 kg. She had gained 6 kg during her pregnancy. She had previously run one hour daily until approximately 12 months before her pregnancy. She denied any drug, alcohol or nicotine consumption. She had thus far had an uncomplicated pregnancy.

**Figure 1:**
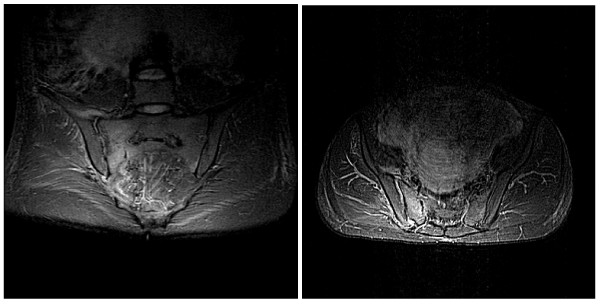
**Coronal and axial short inversion-time inversion-recovery sequence magnetic resonance imaging of the sacrum shows the fracture line of the right lateral mass surrounded by an area of edema**.

Upon admission, a fetal weight of 1,400 g was estimated. Our patient had a temperature of 38.5°C and elevated inflammation parameters, with a C-reactive protein level of 95 mg/L (reference range, < 5 mg/L) and a white blood cell count of 13.6 G/l (reference range 4.3 to 10 G/l). Thrombosis was ruled out by color-coded Doppler ultrasonography. An MRI of her spine and pelvis was obtained. It did not show an ischemic or inflammatory process but did reveal a non-displaced fracture of the right lateral mass of her sacrum with surrounding osseous edema (Figure 1).

Our patient continued to have persistent severe pain despite oral analgesics and immobilization. A Patrick's test was positive and our patient complained of a massive pain in her lower back which increased on direct pressure to the iliosacral joint. She was therefore given epidural anesthesia. She was started on a regimen of ropivacaine 0.2% and sufentanil 0.5 μg/mL. On the second day, the sufentanil was discontinued and she continued to receive ropivacaine only. Thereafter, she experienced significant pain relief. Under regular fetal cardiotocographic monitoring, our patient was then able to mobilize under physiotherapeutic guidance. The remaining course of her pregnancy was uneventful. A qualitative bone density measurement (osteosonometry) performed by her gynecologist one week after delivery showed no signs of pregnancy-related osteoporosis.

At the end of her pregnancy, our patient was completely free from pain. She did not opt for a Cesarean section, as per our recommendation, and delivered a healthy girl weighing 2,950 g by normal spontaneous vaginal delivery at term. In the postpartum period, she could walk with oral analgesics and experienced a clinically uneventful healing of the sacral stress fracture.

## Discussion

The etiology of intrapartum or postpartum sacral stress fractures has thus far not been determined. To date, 29 cases of sacral stress fractures in athletes have been described in the literature [2]. To the best of our knowledge, nine cases of postpartum stress fractures have been described. Of these, only six cases have reported the patient's bone density: five presented with normal and one with decreased bone density [11,12]. The incidence of pregnancy-related osteoporosis is approximately 0.4 cases per 100,000 women. Sacral stress fractures present universally with pain localized to the lower back, sacroiliac region, buttocks or groin. Radicular pain is common and described in a number of cases [2,6]. A positive Patrick's test or a tender sacroiliac joint is typically found on clinical examination. There are several other tests to localize sacroiliac joint pain that are also useful in the examination of sacral stress fracture (Table 1, [13]).

**Table 1:** Specific pain tests for the iliosacral-region from Dreyfuss *et al. *[13]

Test	Sensitivity (%)	Specificity (%)
Pressure to sacral sulcus	95	9
Pain at iliosacral joint	85	8
Pain radiation to the buttock	80	14
Pain at posterior iliac spine	76	47
Bouncing	75	35
Gaenslen's test	71	26
Patrick's test	69	16
Pressure to the center of sacrum	53	29
Thigh thrust	36	50
Pain radiation to the knee	19	63
Pain in sitting position and lifting up the pelvis	3	90

Imaging is paramount in securing the diagnosis. Concerns regarding radiation exposure to the developing fetus limit the imaging options. Plain radiographs expose the unborn child, and are only utilized for ruling out other sources of pain. Shah and Stewart [2] reviewed a series of 27 sacral stress fractures and found 25 cases with normal radiographs.

MRI is the modality of choice during pregnancy because of its lack of radiation emissions. As in our patient, all cases described in the literature involved a vertical fracture line with surrounding edema on the MRI scans. If necessary, after delivery, bone scintigraphy or bone scans with higher sensitivity than MRI can be utilized [2].

Bone density measurements should be performed to rule out pregnancy-related osteoporosis.

Because of the high radiation load, the investigation should occur only after the pregnancy has ended. For assessment of the bone density during pregnancy, osteosonometry can be considered, but its use is controversial and not evidence-based.

Pregnancy-related osteoporosis most often occurs in the third trimester [14,15], and if the bone mineral density does not normalize within five to ten years of the delivery, then the osteoporosis is likely to be permanent [14]. Along with sacral fractures, vertebral compression fractures as well as osteoporosis of the proximal femur are associated with pregnancy. Therefore these areas should be examined for injuries after a bone density measurement.

The first step in the pain management of sacral stress fractures is usually rest and activity modification [2]. This is difficult in pregnant patients in whom the stress on the fracture is exerted by the fetus. There is some controversy with those supporting early mobilization because weight bearing appears to be necessary to stimulate osteoblastic activity [6,11]. In our case of an intrapartum sacral fracture, pain control was the greater challenge because of the limited therapy options. In such cases, we endorse early immobilization until sufficient pain control is achieved. Subsequent partial weight bearing is essential to accelerate bone healing and to reduce other complications of immobility.

The following points should be considered for the application of analgesics during pregnancy: effects on the fetus, stage of the pregnancy, influence on the pregnancy course, influence on the mother, risk of pre-eclampsia and analgesia. In principle, especially early in pregnancy, there should be strict indications, low dosages and a short duration of drug administration. Oral paracetamol is the primary analgesic choice during all phases of pregnancy. Ibuprofen can also be used under a strict maximum daily dose of 1,600 mg. In certain situations, intravenous Perfalgan (paracetamol), morphine or pethidine can be administered. Because of its less systemic side effects, epidural anesthesia is recommended in cases of severe pain of the lower extremity and sacrum that is refractory to other therapies.

The cases currently described in the literature demonstrate that most patients regain their activities of daily living within six weeks. Surgical treatment was never necessary. The heterogeneity of the risk groups (pregnant women, endurance athletes or older patients) and the differences between stress and insufficiency fractures mean that treatment options must be adapted. Nevertheless, in all cases, a prompt diagnosis and sufficient pain management must be achieved.

## Conclusions

During pregnancy, fractures of the sacrum must be included in the differential diagnosis of patients with low back pain. The injury should be diagnosed early, and treatment should be tailored accordingly. Even in the complicated setting of pregnancy, insufficient treatment of severe pain should be avoided.

## Consent

Written informed consent was obtained from the patient for publication of this manuscript and any accompanying images. A copy of the written consent is available for review by the Editor-in-Chief of this journal

## Competing interests

The authors declare that they have no competing interests.

## Authors' contributions

MP analyzed and interpreted the patient data and wrote a major part of the manuscript. RS performed the clinical examination, analyzed the blood levels and partook in the discussion of the manuscript. RP performed the literature review and organized the radiological picture material. PL performed the literature review and completed the abstract. HCP controlled the patient's treatment and managed orthopedic and interdisciplinary treatment. PK interpreted and completed the patient data and wrote a part of the case discussion. All authors read and approved the final manuscript.

## References

[B1] Major NM Helms CA Sacral stress fractures in long-distance runners AJR Am J Roentgenol 2000 174 727 729 10701616 10.2214/ajr.174.3.1740727

[B2] Shah MK Stewart GW Sacral stress fractures: an unusual cause of low back pain in an athlete Spine 2002 27 E104 108 10.1097/00007632-200202150-00023 11840118

[B3] Atwell EA Jackson D Stress fractures of the sacrum in runners: two case reports Am J Sports Med 1991 19 531 533 10.1177/036354659101900523 1741475

[B4] Bottomley M Sacral stress fractures in runners Br J Sports Med 1990 24 243 244 10.1136/bjsm.24.4.243 2097021 PMC1478895

[B5] Crockett HC Wright J Madsen M Bates JE Potter HG Warren RF Sacral stress fractures in an elite basketball player after the use of a jumping machine Am J Sports Med 1999 27 526 528 10424226 10.1177/03635465990270042001

[B6] Lin JT Lachmann E Nagler W Sacral insufficiency fractures: A report of two cases and a review of literature J Womens Health Gend Based Med 2001 10 699 705 10.1089/15246090152563588 11571100

[B7] Sibilia J Javier RM Werle C Kuntz JL Fracture of the sacrum in the absence of osteoporosis of pregnancy: a rare skeletal complication of the postpartum Br J Obstet Gynaecol 1999 106 1096 1097 10.1111/j.1471-0528.1999.tb08121.x 10519439

[B8] Grimaud A Oddo F Thibaud I Brocq O Eller-Ziegler L Fracture of the sacrum caused by bone insufficiency in a pregnant woman J Radiol 1997 78 511 512 9296032

[B9] Lin JT Lutz GE Postpartum sacral fracture presenting as lumbar radiculopathy: a case report Arch Phys Med Rehabil 2004 85 1358 1361 10.1016/j.apmr.2003.09.021 15295766

[B10] Rousiere M Kahan A Job-Deslandre C Postpartal sacral fracture without osteoporosis Joint Bone Spine 2001 68 71 73 10.1016/S1297-319X(01)00262-7 11235785

[B11] Karataş M Başaran C Ozgül E Tarhan C Ağildere AM Postpartum sacral stress fracture: an unusual case of low-back and buttock pain Am J Phys Med Rehabil 2008 87 418 422 10.1097/PHM.0b013e318164a8e6 18303473

[B12] Druga R Drugová B [Postpartal atraumatic sacral fracture. A case report and biomechanical comments] Ceska Gynekol 2008 73 192 195 18646674

[B13] Dreyfuss P Michaelsen M Pauza K McLarty J Bogduk N The value of medical history and physical examination in diagnosing sacroiliac joint pain Spine 1996 21 2594 2602 10.1097/00007632-199611150-00009 8961447

[B14] Nordin BE Roper A Post-pregnancy osteoporosis; a syndrome? Lancet 1955 268 431 434 13234409 10.1016/s0140-6736(55)90214-2

[B15] Kurabayashi T Tamura R Hata Y Nishijima S Tsuneki I Tamura M Yanase T [Secondary osteoporosis update. Bone metabolic change and osteoporosis during pregnancy and lactation] Clin Calcium 2010 20 672 681 20445278

